# Development of Fluorinated Non-Peptidic Ghrelin Receptor Ligands for Potential Use in Molecular Imaging

**DOI:** 10.3390/ijms18040768

**Published:** 2017-04-05

**Authors:** Rareş-Petru Moldovan, Sylvia Els-Heindl, Dennis J. Worm, Torsten Kniess, Michael Kluge, Annette G. Beck-Sickinger, Winnie Deuther-Conrad, Ute Krügel, Peter Brust

**Affiliations:** 1 Helmholtz-Zentrum Dresden-Rossendorf e. V., Institute of Radiopharmaceutical Cancer Research, 04318 Leipzig, Germany; r.moldovan@hzdr.de (R.-P.M.); t.kniess@hzdr.de (T.K.); w.deuther-conrad@hzdr.de (W.D.-C.); 2 Institute of Biochemistry, Universität Leipzig, 04103 Leipzig, Germany; sylvia.els@uni-leipzig.de (S.E.-H.); dennis.worm@uni-leipzig.de (D.J.W.); abeck-sickinger@uni-leipzig.de (A.G.B.-S.); 3 Department of Psychiatry, Universität Leipzig, 04103 Leipzig, Germany; Michael.Kluge@medizin.uni-leipzig.de; 4 Rudolf Boehm Institute of Pharmacology and Toxicology, Medical Faculty, Universität Leipzig, 04107 Leipzig, Germany; ute.kruegel@medizin.uni-leipzig.de

**Keywords:** brain, ghrelin receptor, fluorine, positron emission tomography

## Abstract

The ghrelin receptor (GhrR) is a widely investigated target in several diseases. However, the current knowledge of its role and distribution in the brain is limited. Recently, the small and non-peptidic compound (*S*)-6-(4-bromo-2-fluorophenoxy)-3-((1-isopropylpiperidin-3-yl)methyl)-2-methylpyrido[3,2-d]pyrimidin-4(3*H*)-one ((*S*)-**9**) has been described as a GhrR ligand with high binding affinity. Here, we describe the synthesis of fluorinated derivatives, the in vitro evaluation of their potency as partial agonists and selectivity at GhrRs, and their physicochemical properties. These results identified compounds (*S*)-**9**, (*R*)-**9**, and (*S*)-**16** as suitable parent molecules for ^18^F-labeled positron emission tomography (PET) radiotracers to enable future investigation of GhrR in the brain.

## 1. Introduction

The ghrelin receptor (GhrR), also known as growth hormone secretagogue receptor, is critically involved in various peripheral and central actions of the 28-amino acid orexigenic hormone ghrelin. The peptide, mainly synthesized in the stomach, was identified to stimulate the release of growth hormone from the anterior pituitary [1,2,3,4]. Although thought to be non-expressed in the brain [5,6], occasional findings report otherwise [7,8]. A variety of clinical pharmacological studies demonstrated that ghrelin stimulates appetite, promotes body weight gain, reduces glucose-stimulated insulin secretion, and enhances blood glucose [9,10,11,12,13,14]. The GhrRs are distributed in various peripheral tissues (e.g., adipose tissue, pancreatic islets, thyroid, and myocardium). They contribute to cardiovascular functions and energy homeostasis by regulation of glucose metabolism, lipogenesis, and thermogenesis [7,15,16,17]. They are also widely expressed in various regions of the brain. In the hypothalamic nucleus arcuatus, the GhrR is involved in the regulation of eating behavior [15,18,19,20], and in limbic areas (e.g., hippocampus, ventral tegmentum), it controls the sensitivity of the dopaminergic reward circuit [17,21]. This receptor is constitutively high active, and thereby mediates functions in brain regions usually not accessible for peripheral ghrelin, forms homodimers, and activates Gα_q/11_ proteins [1,7,22,23,24,25,26]. Alternatively, it builds heteromers with other G protein–coupled receptors (GPCRs) (e.g., dopamine D_1_, D_2_, or serotonin 5-HT_2C_ receptors (Rs)). Thereby, it can bias signal transduction via allosteric modulation, which allows dynamic alterations of biological functions [27].

Therefore, in brain areas whose activities are strongly dependent on dopamine signaling, GhrRs have been implicated in the reward circuit [28,29], mood [30], cognitive functions [31,32], and sleep [33], as well as in psychiatric disorders like addiction, depression, and anxiety [34].

Up to now, the relevance of the GhrR in healthy and diseased human brain has been nearly unknown. Apart from the emerging interest to design appetite-stimulating drugs or therapeutics against obesity, there is a demand for radioligands suitable for molecular imaging with positron emission tomography (PET) to investigate alterations of GhrR distribution and kinetics in brain diseases [23,35]. However, due to the peptidic nature of the endogenous ligand, the in vivo imaging of GhrR in the brain with PET remains a challenging task [36,37,38,39].

GhrR selective ligands had already been reported before ghrelin was discovered and identified as an endogenous ligand [40]. Given the peptidic nature of ghrelin, many of the synthetically developed ligands for GhrR possess a peptidic or peptidomimetic motif, mainly structurally related to enkephalin, substance P, and ghrelin itself [5,40]. Though peptidic ligands may enter brain areas next to median eminence via fenestrated capillaries [41], they have a limited access to other areas (e.g., the limbic system) [42].

**Scheme 1:**
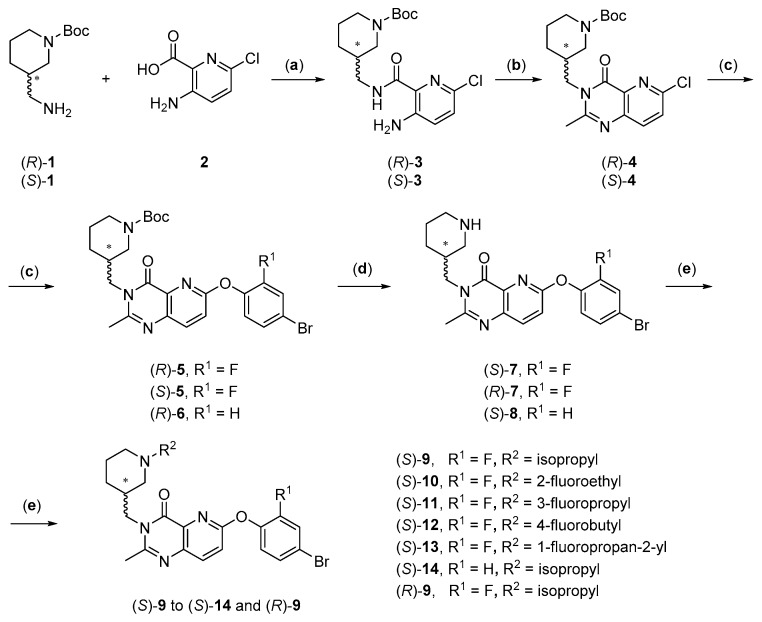
Synthesis of (*S*)-**9** to (*S*)-**14** and (*R*)-**9**. Reagents and conditions: (**a**) BOP, Et_3_N, DCM, room temperature (rt), 20 h, 64%; (**b**) 1,1,1-triethoxy ethane, AcOH, reflux, 3 h, >90%; (**c**) Cs_2_CO_3_, corresponding phenol, DMF, 90 °C, 18 h, 95%; (**d**) TFA/DCM, 1:2, rt, 2 h, quantitative; (**e**) corresponding alkylating agent, K_2_CO_3_, CH_3_CN, 70 °C. BOP, benzotriazole-1-yl-oxy-tris-(dimethylamino)-phosphonium hexafluorophosphate; Et_3_N, triethylamine; DCM, dichloromethane; AcOH, acetic acid; DMF, *N*,*N*-dimethylformamide; TFA, trifluoroacetic acid; DCM, dichloromethane.

To date, a variety of small molecules acting at the GhrR was discovered (for a review see [43]). Therefore, we aimed to develop fluorine-bearing highly affine and selective, non-peptidic ligands with physicochemical properties to cross the blood-brain barrier as lead molecule(s) to establish in the following step an ^18^F-labeled radiotracer for PET qualified for GhrR imaging and quantification. To achieve this, we selected as the lead compound one of the most affine and selective, non-peptidic GhrR ligands reported so far (Scheme 1, (*S*)-**9**, *K*_i_ = 0.01 nM [44]) [45,46]. Compound (*S*)-**9** possesses a fluorine atom at an aromatic position, which offers a challenging late-step incorporation of the ^18^F-label due to the rather low activation for the nucleophilic substitution (S_N_2) reaction. Similar compounds which derived from a common lead molecule as (*S*)-**9** revealed oral bioavailability, brain uptake, and suppressed the ghrelin-mediated insulin secretion in rats [45,47]. Potter et al. also showed favorable pharmacodynamics and brain uptake of a similar, ^11^C-labeled derivative. However, its affinity was not sufficient for in vivo GhrR imaging with PET [36]. With regard to the aspects of late fluorination, the binding and physicochemical studies and the structure-activity analysis, compound (*S*)-**9** and its enantiomer represent suitable surrogate candidates for an ^18^F-labeled GhrR PET radiotracer. Additionally, a new small molecule, (*S*)-**16**, was revealed with comparable binding affinity and reduced partial agonism.

## 2. Results

### 2.1. Chemistry

The lead compound (*S*)-**9** was synthesized as previously described with some modifications [44]. The synthesis started from the commercially available (*R*)-*tert*-butyl 3-(aminomethyl)piperidine-1-carboxylate ((*R*)-**1**, Scheme 1), which was coupled with the carboxylic acid **2** in presence of the coupling reagent BOP (benzotriazole-1-yl-oxy-tris-(dimethylamino)-phosphonium hexafluorophosphate) to give amide (*R*)-**3** that was further cyclized with 1,1,1-triethoxy ethane in thermal reaction conditions to give the 6-methylpirimid-2-one intermediate (*R*)-**4**. Cs_2_CO_3_ promoted diaryl ether formation followed by trifluoroacetic acid (TFA)-mediated *tert*-butyloxycarbonyl (Boc) group removal delivered the key building block (*S*)-**7** in ~55% yield over five steps. *N*-alkylation with 2-iodopropane gave (*S*)-**9** in 89% yield. From a bulk of (*S*)-**7** a small series of fluorinated derivatives ((*S*)-**10**, (*S*)-**11**, (*S*)-**12**, and (*S*)-**13**; see Scheme 1) was synthesized by alkylating the free amino group at the piperidine subunit in basic reaction conditions. In parallel, the hitherto unreported (*R*)-**9** enantiomer was obtained by using the same synthesis sequence (Scheme 1, reactions (a)–(e)) starting from (*S*)-*tert*-butyl 3-(aminomethyl)piperidine-1-carboxylate ((*S*)-**1**). Additionally, to avoid possible radiolysis during the radiofluorination process, compound (*S*)-**14** lacking the fluorine atom at the phenyl subunit was synthesized starting from (*S*)-**4** in >80% yield over three steps (Scheme 1, reactions (c)–(e)).

**Scheme 2:**
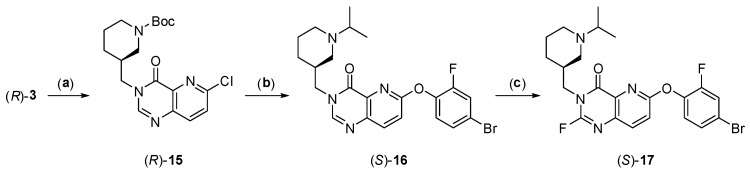
Synthesis of compounds (*S*)-**16** and (*S*)-**17**. Reagents and conditions: (**a**) trimethyl orthoformate, AcOH, reflux, 1 h, >90%; (**b**) i. Cs_2_CO_3_, 2-fluoro-4-bromophenol, DMF, 90 °C, 18 h, 95%; ii. TFA/DCM, 1:2, rt, 2 h, quantitative; iii. corresponding alkylating agent, K_2_CO_3_, CH_3_CN, 70 °C, 89%; (**c**) TBAF, DMF, 130 °C 16 h, 23%. TBAF, tetrabutylammonium fluoride.

Next, we focused on the possibility of introducing a fluorine atom at the pyrimidine 2-position. To achieve this, compound (*R*)-**3** wa*s* cyclized to the corresponding pyrimidine-3(4*H*)-one by trimethyl orthoformate under thermal reaction conditions ((*R*)-**15**, Scheme 2). Diaryl ether formation, Boc-group removal, and piperidine *N*-alkylation with 2-iodopropane were performed as described above to give (*S*)-**16** depicted in Scheme 2. Straight forward fluorination at the aminal position of (*S*)-**16** by tetrabutylammonium fluoride (TBAF) in *N*,*N*-dimethylformamide (DMF) at elevated temperature led to (*S*)-**17** in moderate yield (23%, see Scheme 2).

Further on, together with the lead compound (*S*)-**9**, eight fluorinated compounds (*R*)-**9**, (*S*)-**10**, (*S*)-**11**, (*S*)-**12**, (*S*)-**13**, (*S*)-**14**, (*S*)-**16**, and (*S*)-**17**, are proposed for further structure affinity/activity studies to select candidates for radiolabeling with fluorine-18.

### 2.2. Biological Investigations

#### 2.2.1. Binding Affinities at the Human Ghrelin Receptor (hGhrR)

**Figure 1:**
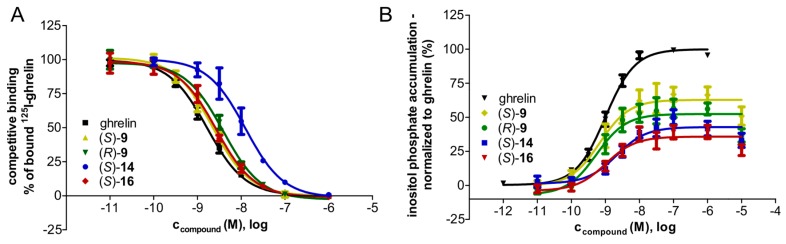
Binding affinity (**A**) and agonistic properties (**B**) of the non-peptidic candidate compounds (*S*)-**9**, (*R*)-**9**, (*S*)-**14**, and (*S*)-**16**. Compounds were tested at membranes of HEK293 cells stably transfected with the hGhrR for competitive binding properties against [^125^I]ghrelin (**A**); Data are means ± SEM (*n* ≥ 4). Agonistic properties of the compounds were analyzed by inositol phosphate accumulation in COS7 cells stably transfected with the hGhrR (**B**). Data were normalized to ghrelin (100% = maximal efficacy, 0% = constitutive receptor activity) and given in percent as means ± SEM of ≥3 independent experiments performed in duplicates.

All the herein newly developed compounds (*R*)-**9**, (*S*)-**10**, (*S*)-**11**, (*S*)-**12**, (*S*)-**13**, (*S*)-**14**, (*S*)-**16**, and (*S*)-**17** together with the previously described compound (*S*)-**9** have been assessed for their binding affinities at the hGhrR using [^125^I-His^9^]ghrelin [48,49] as competitive radioligand. Unlabeled ghrelin served as control with an IC_50_ of 1.7 nM, comparable to previous experiments [50]. (*S*)-**9** was the starting compound for modification with an IC_50_ of 2.2 nM (Figure 1A). A *K*_i_ value of 0.01 nM was previously published for this derivative [44]. However, no *K*_D_ and raw IC_50_ values were given, limiting the comparability of the compound properties. Substitution at the piperidine nitrogen influenced the receptor binding. Compounds (*S*)-**10** and (*S*)-**11** had only low affinity to the receptor, indicated by about 30-fold higher IC_50_ values. Notably, longer substituents resulted in a further increase of the IC_50_ ((*S*)-**12**: 574 nM). Introduction of a fluorine at the isopropyl group ((*S*)-**13**) decreased affinity by about the power of ten. The orientation of the piperidine carbon connected to the azaquinazolinone moiety is of minor importance, as shown by the synthesized (*R*)-**9**, which has only a slightly lower affinity than (*S*)-**9**.

In addition, the significance of the fluorine atom at the aromate and the methyl group at the azaquinazolinone moiety were investigated. Removal of the fluorine in (*S*)-**14** led to a loss of affinity by a factor of about five. Interestingly, whereas removal of the methyl group preserved the binding affinity ((*S*)-**16**), the substitution with fluorine ((*S*)-**17**) did not and caused a severe loss of binding affinity with an IC_50_ of 151 nM (Table 1).

#### 2.2.2. Biological Activity at the hGhrR

Molecules with the highest affinity in the binding assay were tested for their biological activity (Table 2). For that purpose, the inositol phosphate accumulation assay was used [43,44]. The ghrelin receptor was stimulated with different concentrations of the compounds for 2 h in the absence of ghrelin.

Ghrelin served as control with an EC_50_ of 1.0 nM (pEC_50_ = 9.00 ± 0.05, *E*_max_ = 100% ± 3%), which was comparable to data from previous experiments [49,50]. All compounds were found to be partial agonists at the GhrR with similar potencies in the low nanomolar range. (*R*)-**9** and (*S*)-**16** have EC_50_ values of 0.6 and 1.0 nM, respectively, which are similar to the agonistic potency of the starting molecule (*S*)-**9** of 0.7 nM (Table 2). The efficacies of (*S*)-**9**, (*R*)-**9**, (*S*)-**10**, (*S*)-**11** and (*S*)-**13** were all in the same range, as indicated by a maximal receptor activation (*E*_max_) of around 60%.

These data indicate that the stereochemical configuration at the chiral carbon of the piperidine ((*S*)-**9** versus (*R*)-**9**) is neither relevant for the binding affinity nor for the agonistic potency. Importantly, removal of the methyl group at the azaquinazolinone in (*S*)-**16** did not alter the binding affinity compared to (*S*)-**9**, but weakened the partial agonism of the molecule, since (*S*)-**16** showed the lowest agonistic efficacy of all compounds (Figure 1B).

Due to the partial agonism of the compounds in the absence of ghrelin they act as competitive partial antagonists at the GhrR in the presence of ghrelin. As expected, the agonistic efficacies of the compounds correlated with their antagonistic properties. Compound (*S*)-**16** exhibited the strongest antagonistic properties (see supplemental information Table S1 and Figure S1).

#### 2.2.3. Selectivity Studies

The selectivity of the herein reported compounds was assessed by standard screening assays (see methods). As target receptors, the vesicular acetylcholine transporter, the adenosine A_1_Rs and A_2A_Rs, the α7, α4β2, and α3β4 nicotinic acetylcholine receptors (nAChRs), the oxytocin Rs, σ1 and σ2Rs, were investigated. Notably, most of the receptor-specific radioligands used for these assays could not be displaced by the GhrR ligands reported here. Compounds (*S*)-**9** and (*R*)-**9** had a moderate binding affinity to the σ2 receptor (*K*_i_) of 87 ± 23 and 53 ± 7 nM, respectively. Compounds (*S*)-**11** and (*S*)-**12** had a *K*_i_ of 27 ± 8 and 64 ± 14 nM, respectively, at α3β4 nAChRs. Moreover, the most promising GhrR ligand (*S*)-**9** did not show significant responses (≥50% inhibition or stimulation in biochemical assays) at adrenergic α1ARs and α1BRs, chemokine CXCR3Rs, muscarinic M_1_Rs and M_2_Rs, serotonin 5-HT_2B_Rs, and the potassium channel hERG in a commercial screening by Eurofins Panlabs (Taiwan; see Supplementary Materials Table S2).

#### 2.2.4. Distribution Coefficient and Non-Specific Binding

Physicochemical properties of a drug such as lipophilicity and non-specific binding to membranes and plasma proteins are crucial parameters of a potential radiotracer, in particular if it is supposed to pass the blood brain barrier (BBB). The lipophilicity (logD_7.4_) was estimated using the commercially available ACD/Labs-12.5 software and the non-specific binding was estimated by the high-performance liquid chromatography (HPLC)-based hydrophobicity index at immobilized artificial membranes (CHI IAM) [51] for seven fluorine substituted candidate molecules and (*S*)-**13** without fluorine (Table 3).

Since compounds with a logD_7.4_ between 2 and 3 are expected to enter the brain by lipid-mediated free diffusion [52], all compounds presented in this study are suggested to be suitable as brain imaging agents. Further, all investigated ligands meet the requirements of a CHI IAM ≤ 50 as criterion for the non-specific binding of a suitable PET tracer [53].

## 3. Discussion

A large number of medicinal chemistry studies have focused on the development of small molecules acting at the GhrR. Their applicability for therapeutic purposes has been investigated in various preclinical and clinical studies [5,40,43,54]. Apart from that, the present study was aimed at investigating the synthesis of parent compounds which fulfill the pharmacological and physicochemical requirements for brain-penetrating receptor ligands and which provide the possibility to introduce fluorine at a position that enables a late-stage incorporation of the positron emitter ^18^F. In particular, compound (*S*)-**9**, its enantiomer, and the newly synthesized compound (*S*)-**16** meet these criteria.

Compound (*S*)-**9** attracted attention due to its sub-nanomolar affinity to the GhrR combined with optimal pharmacological properties [44,45,46]. It contains a fluorine atom at aromatic position (see Table 1, R^1^). The *ortho*-ether function and the *meta*-bromine atom in respect to the fluorine atom only slightly activate this aromatic position for a putative S_N_2 radiofluorination. However, aromatic radiolabeling is generally difficult and occasionally requires the implementation of highly reactive leaving groups (e.g., ammonium or iodonium salts) and harsh reaction conditions (e.g., high temperature, long reaction time, strong base) [35]. On the other hand, the introduction of the ^18^F radiolabel at aliphatic position is supposed to be more easily performed (see Table 1, *R*^2^).

The modification of synthesis of the lead compound (*S*)-**9** previously described by Hanrahan et al. led to an improved yield of 49% over five steps [44]. The strategy is based on a late-step derivatization using compound (*S*)-**7** as a key building block. The moiety of the piperidine *N*-substituent was investigated using both linear ((*S*)-**10**, (*S*)-**11**, (*S*)-**12**) and branched fluoroalkanes ((*S*)-**13**, see Scheme 1). For comparative in vitro investigation, the (*R*)-**9** enantiomer was synthesized by the same synthesis procedure (Scheme 1). Moreover, the possibility of introducing fluorine at pyrimidine 2-position was proven. The resulting desmethyl derivative (*S*)-**16** was synthesized using trimethyl orthoformate at the cyclization step to (*R*)-**15** (reaction (a), Scheme 2), which was subsequently fluorinated at the aminal position to produce (*S*)-**16** with moderate yield (23%).

In the competitive radioligand binding assay for GhrR, the lead molecule (*S*)-**9** displayed the most promising binding affinity in contrast to molecules with substitution of the isopropyl group at the piperidine subunit. Fluoroethyl, fluoropropyl, and fluorobutyl substitution caused a loss in the GhrR binding affinity (Table 1). The implementation of a 3-fluoro-2-yl group also led to a slightly lower binding affinity. Whereas the substitution of the methyl group by hydrogen at the pyrimidine 2-position (compound (*S*)-**16**) had no effect on binding affinity compared to compound (*S*)-**9**, its substitution with fluorine (compound (*S*)-**17**) impaired this property and made (*S*)-**17** useless as the lead substance for ^18^F labeling. This is probably due to the impact of the highly electronegative fluorine atom on the electron density distribution of the corresponding aromatic ring (see Scheme 2 and Table 1).

Surprisingly, in the case of the (*R*)-**9** enantiomer, the binding affinity was nearly unchanged, unlike the description for similar other compounds [45].

Further characterization of the pharmacological activity of the candidates revealed that (*S*)-**9** and the newly synthesized molecules are highly potent partial agonists at this receptor. This is a new finding, since until now (*S*)-**9** was described solely as a GhrR antagonist [41].

In the additionally performed antagonist experiments, compound (*S*)-**9** had lower partial antagonistic activity than described by Hanrahan et al. [44]. This may be explained by different read-out parameters and cellular systems used in both studies. While the inositol phosphate accumulation serves as an indicator for the Gαq protein-coupled GhrR activity, alterations in the intracellular Ca^2+^ represent a more downstream event, dependent on the inositol phosphate signaling but in addition also on other pathways via Gi and Gβγ proteins [55]. Moreover, results after short incubation times rather reflect kinetic effects and may not allow optimal equilibria conditions between receptor and ligands. This explanation is supported by a study where GSK1614343-elicited effects that were higher in the Ca^2+^ assay compared to the inositol phosphate accumulation assay [56]. It can be assumed that potential side effects by competitive antagonism of these compounds would affect the GhrR function only in a few regions easily accessible for ghrelin, such as the arcuate nucleus.

The non-occupied GhrR possesses constitutive activity in various brain regions (including hippocampus and amygdala) [42]. Hence in the absence of ghrelin, the effects of the potential ligands on the receptor should be also considered for (*S*)-**9** and the newly synthesized compounds. Indeed, a higher activity GhrR was observed by the addition of compounds (*S*)-**9** and (*R*)-**9** at a concentration of 10^−5^ M. Similar agonistic effects were described for GSK1614343, another non-peptidic ligand [57].

With regard to PET imaging, radiolabeled non-peptidic GhrR ligands are expected to have virtually no effects on the function of the target. The radiolabel and the high sensitivity of its detection allow the use of concentrations which are much lower than those needed to elicit any functional effect [35]. Although agonistic or antagonistic properties are very important for therapeutic drugs, this is not applicable for PET tracers.

Potential side effects of GhrR PET tracers are unlikely related to the receptor function but to potential high-affinity binding to other targets which would impair the quantitation of the receptor parameters. The most promising compound (*S*)-**9** did not show significant binding at adrenergic α1ARs and α1BRs, chemokine CXCR3Rs, muscarinic M1Rs and M2Rs, serotonin 5-HT2BRs, and the potassium channel hERG. Also, the σ2 receptor binding (*K*_i_ = 87 ± 23 nM) is obviously too low to be of any influence on PET imaging [35].

All new GhrR ligands are expected to cross the BBB, as indicated by their LogD_7.4_ values. The derivatives with a linear fluoroalkyl group ((*S*)-**11** and (*S*)-**12**) and in particular the fluoroethyl substituted ((*S*)-**10**) had the highest LogD, and thereby may be less appropriate as potential brain imaging agent. On the other hand, for (*S*)-**10** the lowest non-specific binding in vivo is predicted. This suggests that membrane permeability and non-specific binding are not necessarily correlated. Notably, (*S*)-**9**, *(R*)-**9**, and (*S*)-**16** have favorable physiochemical properties for brain penetration and low non-specific binding, and are therefore the three most applicable compounds of this study for future radiolabeling. The introduction of the ^18^F-isotope at any of the target molecules is possible by the nucleophilic (S_N_2) substitution of a suitable leaving group (e.g., nitro, iodonium, or ammonium salt), as previously described [35]. The implementation of the leaving group at the aryl position of (*S*)-**9** or (*S*)-**16** can be performed by the synthetic strategy described in Scheme 1 and Scheme 2, respectively (e.g., Scheme 1 R^1^ = isopropyl, R^2^ = NO_2_).

In summary, a novel small series of fluorinated GhrR ligands was synthesized by modifying the structure of compound (*S*)-**9**. While fluoro derivatizations performed at the *N*-piperidine and pirimidine-2-position led to a decrease in the GhrR affinity, the enantiomer (*R*)-**9** showed high GhrR binding in the low nanomolar range. The physicochemical evaluation suggests that all GhrR ligands are able to cross the BBB and to exhibit low non-specific binding. Furthermore, the compounds are of high selectivity towards the GhrR. These findings qualify compounds (*S*)-**9**, its enantiomer, and (*S*)-**16** as suitable starting molecules for the development of an ^18^F-labeled GhrR PET tracer for brain imaging.

## 4. Materials and Methods

### 4.1. General Methods

Unless otherwise noted, moisture sensitive reactions were conducted under dry nitrogen or argon. All chemicals and reagents were purchased from commercially available sources and used without further purification. Thin-layer chromatography (TLC): Silica gel 60 F254 plates (Merck KGaA, Darmstadt, Germany). Flash chromatography (fc): Silica gel 60, 40–64 µm (Merck). Room temperature (rt) was 21 °C. Mass spectrometry (MS): MAT GCQ (Thermo Finnigan MAT GmbH, Bremen, Germany). ^1^H, ^13^C spectra were recorded on VARIAN “MERCURY plus” (300 MHz for ^1^H NMR, 75 MHz for ^13^C NMR) and VARIAN “MERCURY plus” and BRUKER DRX-400 (400 MHz for ^1^H NMR, 100 MHz for ^13^C NMR, 377 MHz); δ in ppm related to tetramethylsilane; coupling constants (*J*) are given with 0.1 Hz resolution. Multiplicities of NMR signals are indicated as follows: s (singlet), d (doublet), t (triplet), m (multiplet), dd (doublet of doublets), dt (doublet of triplets). ESI/Ion trap mass spectra (LRMS) were recorded with a Bruker Esquire 3000 plus instrument (Bruker Corporation, Billerica, MA, USA). High resolution mass spectra were recorded on a FT-ICR APEX II spectrometer (Bruker Daltonics; Bruker Corporation) using electrospray ionization (ESI) in positive ion mode. Lipophilicity at pH = 7.4 (cLogD_7.4_) was calculated using ACD/Labs-12.5.

### 4.2. Procedures and Compound Characterization

*tert*-Butyl (*R*)-3-((3-amino-6-chloropicolinamido)methyl)piperidine-1-carboxylate ((*R*)-**3**). General Procedure 1. BOP (3.1 g, 1.2 equiv, 6.9 mmol) and Et_3_N (1.2 mL, 3 equiv, 17.4 mmol) were added to a solution of amine **1** (1.6 g, 1.3 equiv, 7.5 mmol) and acid **2** (1 g, 1 equiv, 5.8 mmol) in 25 mL DCM, and the reaction mixture was stirred at rt for 20 h. After completion, the NaHCO_3_ aqueous was added (20 mL) and the whole was extracted with 2 × 20 mL DCM. Drying over Na_2_SO_4_ followed by solvent evaporation under reduced pressure afforded a thick oil which was purified by flash chromatography (silica, ethyl acetate (EA)/petroleum ether (40–65 °C) (IH), 1:4 to 1:1) to give (*R*)-**3** (1.3 g, 64% yield) as colorless solid.

*tert*-Butyl (*R*)-3-((6-chloro-2-methyl-4-oxopyrido[3,2-d]pyrimidin-3(4*H*)-yl)methyl)piperidine-1-carboxylate ((*R*)-**4**). General Procedure 2. Compound **3** (1 g, 1 equiv, 2.6 mmol), in 1.1.1-triethoxy ethane (2 mL, 5 equiv, 13 mmol) and AcOH (170 µL, 1 equiv, 2.6 mmol) was warmed for 3 h at 135 °C. Upon completion (TLC), the solvent was removed under reduced pressure and the residue was purified by flash chromatography (silica, EA) to give (*R*)-**4** (960 mg, 90% yield) as tan solid.

*tert*-Butyl (*R*)-3-((6-(4-bromo-2-fluorophenoxy)-2-methyl-4-oxopyrido[3,2-d]pyrimidin-3(4*H*)-yl)methyl)piperidine-1-carboxylate ((*R*)-**5**). General Procedure 3. 4-Bromo-2-fluorophenol (370 mg, 3 equiv, 3.8 mmol) was added to a solution of (*R*)-**4** (500 mg, 1 equiv, 1.3 mmol) and Cs_2_CO_3_ (1.2 g, 3 equiv, 3.8 mmol) in 5 mL DMF, and the reaction was performed at 90 °C overnight. An aqueous solution of NaHCO_3_ (20 mL) was added and the mixture was extracted with DCM (2 × 20 mL). Drying over Na_2_SO_4_ and solvent elimination gave a solid which was purified by flash chromatography (silica, EA) to give (*R*)-**5** (630 mg, 90% yield) as white solid.

(*S*)-6-(4-Bromo-2-fluorophenoxy)-2-methyl-3-(piperidin-3-ylmethyl)pyrido[3,2-d]pyrimidin-4(3*H*)-one). General Procedure 4. The Boc-protected (*R*)-**5** (900 mg, 1.6 mmol) was dissolved in 2 mL TFA/DCM (1:2) and stirred at rt for 2 h. Elimination of the solvent gave a thick oil which was diluted with 10 mL DCM and washed with 10 mL NaHCO_3_ aqueous solution. Drying over Na_2_SO_4_ followed by solvent evaporation under reduced pressure gave (*S*)-**7** as white tan solid in quantitative yield.

(*S*)-6-(4-Bromo-2-fluorophenoxy)-3-((1-isopropylpiperidin-3-yl)methyl)-2-methylpyrido[3,2-d]pyrimidin-4(3*H*)-one ((*S*)-**9**) General Procedure 5. 2-iodopropane (30 µL, 3 equiv, 0.3 mmol) was added to a solution of (*S*)-**7** (44.7 mg, 1 equiv, 0.1 mmol) and K_2_CO_3_ (41.4 mg, 3 equiv, 0.3 mmol) in CH_3_CN (2 mL), and the reaction was performed at 70 °C overnight. H_2_O (10 mL) was then added at room temperature and the mixture was extracted with DCM (2 × 10 mL). The combined organic phases were dried over Na_2_SO_4_, the solvent was evaporated, and the resulting residue was purified by flash chromatography (silica, CHCl_3_/MeOH, 9:1) to give (*S*)-**9** (43 mg, 89%yield) as white solid. ^1^H NMR (400 MHz, CDCl_3_): *δ* (ppm) = 8.01 (d, *J* = 8.8 Hz, 1H), 7.45–7.14 (m, 4H), 4.22 (m, 1H), 3.93 (m, 1H), 3.47–3.28 (m, 2H), 3.21 (d, *J* = 11.2 Hz, 1H), 3.04 (m, 1H), 2.85–2.52 (m, 2H), 2.72 (s, 3H), 2.34–2.33 (m, 1H), 2.00–1.88 (m, 3H), 1.40 (d, *J* = 6.6 Hz, 3H), 1.32 (d, *J* = 6.6 Hz, 3H). ^13^C NMR (100 MHz, CDCl_3_): *δ* (ppm) = 60.6, 160.3, 155.5, 154.0, 153.0, 141.6, 139.9 (d, *J* = 10.1 Hz), 139.7, 133.6, 127.7 (d, *J* = 3.8 Hz), 124.9, 120.4 (d, *J* = 21.6 Hz), 118.6, 117.8 (d, *J* = 8.3 Hz), 57.7, 52.8, 47.3, 34.3, 32.2, 27.4, 23.1, 22.1, 17.4, 16.3. HRMS (ESI+): *m*/*z* (%) = 489.1293, calcd. 489.1296 [M + H]^+^.

(*S*)-6-(4-Bromo-2-fluorophenoxy)-3-((1-(2-fluoroethyl)piperidin-3-yl)methyl)-2-methylpyrido[3,2-d]pyrimidin-4(3*H*)-one ((*S*)-**10**) was obtained according to General Procedure 5. ^1^H NMR (400 MHz, CDCl_3_): *δ* (ppm) = 7.99 (d, *J* = 8.8 Hz, 1H), 7.48–7.12 (m, 4H), 4.89–4.41 (m, 2H), 4.14 (d, *J* = 7.8 Hz, 1H), 3.96 (s, 1H), 2.90 (dd, *J* = 57.0, 36.6 Hz, 4H), 2.69 (s, 3H), 2.47 (d, *J* = 47.8 Hz, 3H), 1.98–1.63 (m, 3H), 1.31 (d, *J* = 8.8 Hz, 1H). ^13^C NMR (100 MHz, CDCl_3_): *δ* (ppm) = 160.3 (d, *J* = 7.4 Hz), 155.6, 154.1, 153.1, 141.5, 139.9 (d, *J* = 12.0 Hz), 139.5, 133.8, 127.8 (d, *J* = 3.7 Hz), 125.0, 120.5 (d, *J* = 21.5 Hz), 118.6, 117.9 (d, *J* = 8.2 Hz), 58.2 (d, *J* = 20.2 Hz), 57.4, 53.7, 47.6, 35.3, 27.7, 27.6, 23.9, 23.2. HRMS (ESI+): *m*/*z* (%) = 493.1049, calcd. 493.1045 [M + H]^+^.

(*S*)-6-(4-Bromo-2-fluorophenoxy)-3-((1-(3-fluoropropyl)piperidin-3-yl)methyl)-2-methylpyrido[3,2-d]pyrimidin-4(3*H*)-one ((*S*)-**11**) was obtained according to General Procedure 5. ^1^H NMR (400 MHz, CDCl_3_): *δ* (ppm) = 7.98 (d, *J* = 8.8 Hz, 1H), 7.51–7.16 (m, 4H), 4.64–4.48 (m, 1H), 4.48–4.34 (m, 1H), 4.18 (m, 1H), 3.93 (m, 1H), 3.39–2.88 (m, 2H), 2.76 (m, 2H), 2.67 (s, 3H), 2.70–2.25 (m, 2H), 2.22–1.82 (m, 3H) 1.83 (m, 2H), 1.50–1.12 (m, 2H). ^13^C NMR (100 MHz, CDCl_3_): *δ* (ppm) = 160.4, 160.3, 155.6, 154.1, 153.1, 141.5, 139.9 (d, *J* = 12.0 Hz), 139.5, 133.7, 127.8 (d, *J* = 3.7 Hz), 125.1, 120.6, 120.4, 118.6, 117.9 (d, *J* = 8.2 Hz), 64.8, 57.1, 54.7, 53.1, 47.6, 40.2, 34.8, 27.7, 26.4, 26.1, 23.2. HRMS (ESI+): *m*/*z* (%) = 507.1205, calcd. 507.1202 [M + H]^+^.

(*S*)-6-(4-Bromo-2-fluorophenoxy)-3-((1-(4-fluorobutyl)piperidin-3-yl)methyl)-2-methylpyrido[3,2-d]pyrimidin-4(3*H*)-one ((*S*)-**12**) was obtained according to General Procedure 5. ^1^H NMR (400 MHz, CDCl_3_): *δ* (ppm) = 7.99 (d, *J* = 8.8 Hz, 1H), 7.56–6.98 (m, 4H), 4.49 (t, *J* = 5.8 Hz, 1H), 4.38 (t, *J* = 5.3 Hz, 1H), 4.25–3.85 (m, 2H), 2.79 (m, 2H), 2.68 (s, 3H), 2.46 (m, 2H), 2.29 (s, 1H), 2.09 (m, 2H), 1.84–1.52 (m, 7H), 1.26 (m, 1H). ^13^C NMR (100 MHz, CDCl_3_): *δ* (ppm) = 160.2, 154.3 (t, *J* = 126.9 Hz), 141.4, 139.9 (d, *J* = 11.9 Hz), 139.4, 133.9, 127.7 (d, *J* = 3.8 Hz), 124.9 (d, *J* = 1.5 Hz), 120.5, 120.3, 118.4, 117.8, 83.7 (d, *J* = 164.7 Hz), 58.2, 57.5, 53.2, 48.0, 35.6, 28.3, 28.3, 28.1, 24.6, 24.0, 22.2. HRMS (ESI+): *m*/*z* (%) = 521.1355, calcd. 521.1358 [M + H]^+^.

6-(4-Bromo-2-fluorophenoxy)-3-(((3*S*)-1-(1-fluoropropan-2-yl)piperidin-3-yl)methyl)-2-methylpyrido[3,2-d]pyrimidin-4(3*H*)-one ((*S*)-**13**) was obtained according to General Procedure 5. ^1^H NMR (400 MHz, CDCl_3_): *δ* (ppm) = 7.98 (d, *J* = 8.8 Hz, 1H), 7.46–7.11 (m, 4H), 4.29–3.86 (m, 2H), 3.86–3.28 (m, 1H), 3.20–2.88 (m, 2H), 2.75–2.55 (m, 1H), 2.68 (s, 3H), 2.50 (m, 1H), 2.28 (m, 2H), 1.93–1.67 (m, 3H), 1.38–1.22 (m, 2H), 1.16 (t, *J* = 7.2 Hz, 3H). ^13^C NMR (100 MHz, CDCl_3_): *δ* (ppm) = 160.4 (d, *J* = 16.1 Hz), 154.4 (t, *J* = 127.0 Hz), 141.6, 140.0 (d, *J* = 12.1 Hz), 139.6, 133.8, 128.9, 127.9 (d, *J* = 3.7 Hz), 125.8, 125.1, 120.6 (d, *J* = 21.5 Hz), 118.7, 118.0 (d, *J* = 8.2 Hz), 56.5, 52.6, 52.5, 47.7, 35.3, 29.8, 27.9, 23.4, 23.2, 10.8. HRMS (ESI+): *m*/*z* (%) = 507.1203, calcd. 507.1202 [M + H]^+^.

(*R*)-6-(4-Bromo-2-fluorophenoxy)-3-((1-isopropylpiperidin-3-yl)methyl)-2-methylpyrido[3,2-d]pyrimidin-4(3*H*)-one ((*R*)-**9**) was obtained according to General Procedures 1–5. ^1^H NMR (400 MHz, CDCl_3_): *δ* (ppm) = 8.01 (d, *J* = 8.8 Hz, 1H), 7.45–7.14 (m, 4H), 4.21 (s, 1H), 3.93 (m, 1H), 3.45–3.25 (m, 2H), 3.25–2.95 (m, 2H), 2.86–2.49 (m, 2H), 2.73 (s, 3H), 2.34–2.33 (m, 1H), 2.00–1.88 (m, 3H), 1.40 (d, *J* = 6.6 Hz, 3H), 1.32 (d, *J* = 6.6 Hz, 3H). ^13^C NMR (100 MHz, CDCl_3_): *δ* (ppm) = 160.7, 160.3, 155.6, 154.0, 141.6, 140.0 (d, *J* = 12.0 Hz), 139.7, 133.6, 127.8 (d, *J* = 3.7 Hz), 124.9, 120.5 (d, *J* = 21.6 Hz), 118.7, 117.9 (d, *J* = 8.3 Hz), 58.0, 52.8, 47.4, 47.0, 34.2, 27.3, 23.1, 22.0, 17.3, 16.3. HRMS (ESI+): *m*/*z* (%) = 489.1292, calcd. 489.1296 [M + H]^+^.

(*S*)-6-(4-Bromo-2-fluorophenoxy)-3-((1-isopropylpiperidin-3-yl)methyl)pyrido[3,2-d]pyrimidin-4(3*H*)-one ((*S*)-**16**) was obtained according to General Procedures 1–5. ^1^H NMR (400 MHz, CDCl_3_): *δ* (ppm) = 8.18–7.89 (m, 2H), 7.62–7.13 (m, 4H), 4.00 (d, *J* = 6.9 Hz, 2H), 2.79–2.74 (m, 3H), 2.38–2.70 (m, 3H), 1.92–1.53 (m, 3H), 1.26 (m, 1H), 1.08 (t, *J* = 6.7 Hz, 6H). ^13^C NMR (100 MHz, CDCl_3_): *δ* (ppm) = 160.6, 159.1, 155.6, 153.0, 146.4, 142.3, 140.0, 135.7, 127.7 (d, *J* = 3.5 Hz), 124.9, 120.4 (d, *J* = 21.6 Hz), 118.3, 118.0 (d, *J* = 8.3 Hz), 55.4, 52.0, 49.7, 48.6, 35.1, 27.9, 23.5, 17.9, 17.4. HRMS (ESI+): *m*/*z* (%) = 475.1139, calcd. 475.1139 [M + H]^+^.

(*S*)-6-(4-Bromo-2-fluorophenoxy)-2-fluoro-3-((1-isopropylpiperidin-3-yl)methyl)pyrido[3,2-d]pyrimidin-4(3*H*)-one ((*S*)-**17**). TBAF (87 mg, 2 equiv, 0.33 mmol) was added to a solution of (*S*)-**16**) (80 mg, 1 equiv, 0.16 mmol) in 2 mL DMF and the reaction mixture was heated to 130 °C for 16 h. Upon cooling, water (5 mL) was added and the solution was extracted with DCM (2 × 5 mL). Drying over Na_2_SO_4_ followed by solvent elimination gave an oil which was purified by flash chromatography (silica, CH_2_Cl_2_/MeOH, 18:1 to 9:1) to give (*S*)-**17**) (19 mg, 23% yield) as light brown solid. ^1^H NMR (400 MHz, CDCl_3_): *δ* (ppm) = 7.71 (s, 1H), 7.54–7.34 (m, 2H), 7.18 (dd, *J* = 14.9, 8.7 Hz, 2H), 3.26 (m, 5H), 2.35 (m, 4H), 1.86 (m, 2H), 1.36 (t, *J* = 6.9 Hz, 6H), 1.03 (m, 1H). ^13^C NMR (100 MHz, CDCl_3_): *δ* (ppm) = 166.5, 159.6, 155.8, 155.6 (d, *J* = 25.7 Hz), 139.92 (d, *J* = 12.1 Hz), 134.5, 133.6, 129.1, 128.1 (d, *J* = 3.6 Hz), 125.2, 121.0, 120.4 (d, *J* = 21.4 Hz), 118.1 (d, *J* = 8.2 Hz), 115.8, 57.6, 51.8, 48.4, 41.8, 29.7, 27.0, 24.0, 19.7, 15.5. HRMS (ESI+): *m*/*z* (%) = 493.1041, calcd. 493.1045 [M + H]^+^.

### 4.3. Biology In Vitro Characterization of Compounds

#### 4.3.1. Radioligand Binding Assay

Two micrograms of human GhrR membranes (prepared from HEK293 cells stably expressing the fusion protein hGhrR-eYFP), 60 pM [^125^I-His^9^]ghrelin (PerkinElmer, Waltham, MA, USA), and different concentrations of the test compound were incubated in HEPES buffer (20 mM HEPES, 20 mM CaCl_2_, 0.8 mM MgCl_2_, 1% Pefabloc, 1% bovine serum albumin, pH 7.4) in a 96-well plate on a shaker (200 rpm) for 2.5 h at rt. Each concentration was tested in duplicate. The membrane-bound radioligand was trapped on a Filtermat B (PerkinElmer) pre-soaked with 0.1% polyethylenimine in H_2_O by using a filtermat harvester (PerkinElmer). After drying for 20 min at 55 °C, a Meltilex scintillation sheet (PerkinElmer) was melted onto the filtermat and the samples were counted in a Microbeta scintillation counter (PerkinElmer). For each compound, all data points (*n* = 4–6) from at least two independent experiments were combined and IC_50_ values were obtained by a sigmoidal dose-response fit. Data were normalized to no competition (100%, minimal ghrelin effect) and full competition (0%, maximal ghrelin effect).

#### 4.3.2. Inositol Phosphate Accumulation Assay

Briefly, 60,000 to 80,000 cells/well of COS7 cells, stably expressing the fusion protein hGhrR-eYFP, were seeded in 48-well plates in Dulbecco’s modified Eagle’s medium (DMEM) with 10% fetal calf serum (FCS) and 0.4 mg/mL hygromycin B. The next day, the cells were labeled with [2-^3^H]-myo-inositol (2 µCi/mL, PerkinElmer). Stimulation with different concentrations of the compounds alone or with 10^−8^ M ghrelin together with different concentrations of the compounds (antagonist experiments) was performed in duplicates for 2 h [58]. Cell debris was removed and the supernatant purified on an anion-exchange resin (BioRad, Hercules, CA, USA). The obtained data were analyzed with GraphPad Prism 5.0 (GraphPad Software, San Diego, CA, USA). Dpm (decay per minute) values were normalized to ghrelin effects (100% = maximal efficacy, 0% = constitutive receptor activity). EC_50_/IC_50_ values were calculated from at least three independent experiments. *E*_max_ is defined as the difference between maximal and minimal effect in presence of the compound.

#### 4.3.3. Determination of CHI IAM Values

The chromatographic hydrophobicity index on immobilized artificial membranes (CHI IAM) was experimentally determined by a HPLC method described by Valko and co-workers [51] using a IAM PC DD2 column (100 × 4.6 mm, 10 µM, Regis Technologies, IL, USA), a gradient of acetonitrile, and 50 mM ammonium acetate as eluent and acetanilide, acetophenone, 1,4-dinitrobenzene, anisole propiophenone, valerophenone, and octanophenone as references.

#### 4.3.4. Selectivity Studies

All compounds were investigated regarding their potential to bind to rat σ1 and σ2 receptors, human α4β2, α3β4, and α7 nAChRs, human A_1_ and A_2A_ receptors, human oxytocin receptor, and rat VAChT at 100 nM and 1 µM by radioligand displacement studies using the following conditions:

(a) rat σ1 receptor = rat brain homogenate, [^3^H]pentazocine, 50 mM TRIS-HCl, pH 7.4, 120 mM NaCl, 5 mM KCl, 2 mM CaCl_2_, 1 mM MgCl_2_, 120 min incubation at rt;
(b) rat σ2 receptor = rat liver homogenate, [^3^H]DTG + 1 µM dextrallorphan, 50 mM TRIS-HCl, pH 7.4, 120 mM NaCl, 5 mM KCl, 2 mM CaCl_2_, 1 mM MgCl_2_, 120 min incubation at rt;
(c) rat VAChT = homogenates of transfected PC12 cells (by courtesy of Ali Roghani, Texas Tech University, Lubbock, TX, USA), [^3^H]vesamicol, 50 mM TRIS-HCl, pH 7.4, 120 mM NaCl, 5 mM KCl, 2 mM CaCl_2_, 1 mM MgCl_2_, 60 min incubation at rt;
(d) human oxytocin receptor = homogenates of transfected CHO cells (by courtesy of Bice Chini, Isituto di Neuroscienze, Milano, Italy), [^3^H]oxytocin, 50 mM TRIS-HCl, pH 7.4, 5 mM MgCl_2_, 0.1% BSA, 60 min incubation at rt;
(e) human nAChR = homogenates of transfected HEK (α4β2, α3β4) and SH-SY5Y (α7) cells (by courtesy of Dominik Feuerbach, Novartis, Basel, Switzerland), [^3^H]epibatidine (α4β2, α3β4) or [^3^H]MLA (α7), 50 mM TRIS-HCl, pH 7.4, 120 mM NaCl, 5 mM KCl, 2 mM CaCl_2_, 1 mM MgCl_2_, 60 min incubation at rt;
(f) human adenosine receptors = homogenates of transfected CHO cells (by courtesy of Karl-Norbert Klotz, Julius-Maximilians-Universität, Würzburg, Germany), [^3^H]DPCPX (A1) or [^3^H]ZM241385 (A2A), 50 mM TRIS-HCl, pH 7.4, 1 mM MgCl_2_ (A1) or 50 mM TRIS-HCl, pH 7.4, 100 mM NaCl, 5 mM MgCl_2_, 1 M EDTA (A2A), 120 min (A1) or 60 min (A2A) incubation at rt.

The incubations were terminated by rapid filtration via glass-fiber filters presoaked in PEI (0.3%, 60 min at rt), and the filter-bound radioactivity was counted by liquid scintillation counting (Tricarb, PerkinElmer).

For compounds displacing >50% of the radioligand at 1 µM or 100 nM, *K*_i_ values were determined by investigating seven concentrations of the test compound (0.01 nM to 10 µM) as described.

## Figures and Tables

**Table 1:** Competitive binding assay at HK293 cell membranes expressing hGhrR (human ghrelin receptor) with [^125^I-His^9^]ghrelin.

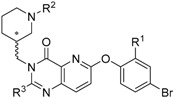						
Compound	R^1^	R^2^	R^3^	MW	IC_50_ [nM]	pIC_50_^1^
Ghrelin					1.7	8.78 ± 0.04
(*S*)-**9**	F	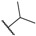	Me	489	2.2	8.66 ± 0.05
(*R*)-**9**	F	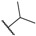	Me	489	3.9	8.41 ± 0.05
(*S*)-**10**	F	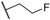	Me	493	69	7.16 ± 0.11
(*S*)-**11**	F	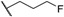	Me	507	66	7.18 ± 0.09
(*S*)-**12**	F	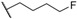	Me	521	574	6.24 ± 0.12
(*S*)-**13**	F	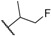	Me	507	13	7.90 ± 0.13
(*S*)-**14**	H	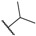	Me	471	12	7.91 ± 0.10
(*S*)-**16**	F	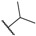	H	475	2.7	8.57 ± 0.06
(*S*)-**17**	F	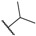	F	493	151	6.82 ± 0.09

^1^ Mean values ± SEM (*n* ≥ 4).

**Table 2:** Inositol phosphate accumulation assay to determine agonistic potencies of candidate molecules.

Compound	EC_50_ (nM)	pEC_50_^1^	*E*_max_^1^ (%) ^2^	*n* ^3^
(*S*)-**9**	0.7	9.17 ± 0.19	63 ± 6	≥3
(*R*)-**9**	0.6	9.26 ± 0.21	59 ± 6	≥3
(*S*)-**10**	1.6	8.80 ± 0.14	64 ± 5	≥3
(*S*)-**11**	2.9	8.54 ± 0.15	58 ± 4	≥3
(*S*)-**13**	1.4	8.87 ± 0.13	57 ± 4	≥3
(*S*)-**14**	2.1	8.67 ± 0.20	42 ± 4	≥3
(*S*)-**16**	1.0	8.99 ± 0.20	40 ± 4	≥3

^1^ Mean values ± SEM; ^2^ data refer to ghrelin with *E*_max_ as 100%; ^3^ number of independent experiments in duplicates. EC_50_, Half maximal effective concentration.

**Table 3:** Calculated logD_7.4_ values and experimentally determined CHI IAM (chromatographic hydrophobicity index on immobilized artificial membrane) values of GhrR ligands.

Compound	cLogD_7.4_^1^	CHI IAM
(*S*)-**9**	2.1	43.0
(*R*)-**9**	2.1	42.8
(*S*)-**10**	3.4	39.6
(*S*)-**11**	3.1	42.2
(*S*)-**12**	3.0	43.3
(*S*)-**13**	3.1	42.8
(*S*)-**14**	2.4	42.8
(*S*)-**16**	2.9	42.3

^1^ calculated with ACD/Labs-12.5.

## Supplementary Materials

Supplementary materials can be found at www.mdpi.com/1422-0067/18/4/768/s1.

## Abbreviations

AcOH	Acetic acid
GhrR	Ghrelin receptor
BOP	Benzotriazole-1-yl-oxy-tris-(dimethylamino)-phosphonium hexafluorophosphate
BBB	Blood-brain barrier
Boc	*tert*-Butyloxycarbonyl
CHI IAM	Chromatographic hydrophobicity index on immobilized artificial membranes
DCM	Dichloromethane
DMF	*N*,*N*-Dimethylformamide
EA	Ethyl acetate
HPES	4-(2-Hydroxyethyl)-1-piperazineethanesulfonic acid
HPLC	High-performance liquid chromatography
HRMS	High resolution mass spectrometry
IC_50_	The half maximal inhibitory concentration
IH	Petroleum ether (40–65 °C)
M	Molarity
MW	Molecular weight
PET	Positron emission tomography
SPECT	Single-photon emission computed tomography
TBAF	Tetrabutylammonium fluoride
Et_3_N	Triethylamine
TFA	Trifluoroacetic acid
