# Supplementary material for: Development of Fluorinated Non-Peptidic Ghrelin Receptor Ligands for Potential Use in Molecular Imaging
Source: Int J Mol Sci. 2017 Apr 5;18(4):768. doi: 10.3390/ijms18040768 (PMC5412352; doi:10.3390/ijms18040768)
Supplement: Supplementary file 1 [file ijms-18-00768-s001.pdf]

Article

Development of Fluorinated Non-Peptidic Ghrelin Receptor Ligands for Potential Use in Molecular Imaging

Rareş-Petru Moldovan, Sylvia Els-Heindl, Dennis J. Worm, Torsten Kniess, Michael Kluge, Annette G. Beck-Sickinger, Winnie Deuther-Conrad, Ute Krügel, and Peter Brust

Supplementary Materials

Antagonistic properties at the hGhrR

Molecules with the highest affinity in the binding assay were additionally tested for their antagonistic potency in an inositol phosphate accumulation assay [1] (Table S1). The receptor was first pre-stimulated with different concentrations of the compounds for 5 min and then 10^{-8} M ghrelin was added.

Table S1. Inositol phosphate accumulation assay to determine antagonistic potencies of candidate molecules with 10^{-8} M ghrelin.

Compound	IC_{50} (nM)	pIC_{50}[1]	E_{max}[1] (%)	n[2]
(*S*)-**9**	24.8	7.61 ± 0.20	38 ± 4	≥ 3
(*R*)-**9**	17.5	7.76 ± 0.24	45 ± 5	4
(*S*)-**10**	345	6.46 ± 0.48	22 ± 4	≥ 3
(*S*)-**11**	349	6.46 ± 0.29	56 ± 10	4
(*S*)-**13**	207	6.69 ± 0.29	31 ± 5	5
(*S*)-**14**	123	6.91 ± 0.18	57 ± 6	4
(*S*)-**16**	28.7	7.54 ± 0.15	65 ± 5	5

[1] Mean values ± SEM, [2] number of independent experiments in duplicates

Ghrelin was used as control with an EC_{50} of 0.9 nM (pEC_{50} = 9.05 ± 0.06, E_{max} = 100 ± 3%). Due to the partial agonism of the compounds, the effect of ghrelin cannot be completely antagonized. The assay provided IC_{50} values for (*R*)-**9** and (*S*)-**16** of 17.5 nM and 28.7 nM, respectively, which were similar to the antagonistic potency of the starting molecule (*S*)-**9** of 24.8 nM. (*S*)-**10**, (*S*)-**11**, (*S*)-**14,** and (*S*)-**13** were much less potent, as indicated by their IC_{50} values (Table S1). The highest antagonistic efficacy could be observed for (*S*)-**16** (E_{max} of 65 %).

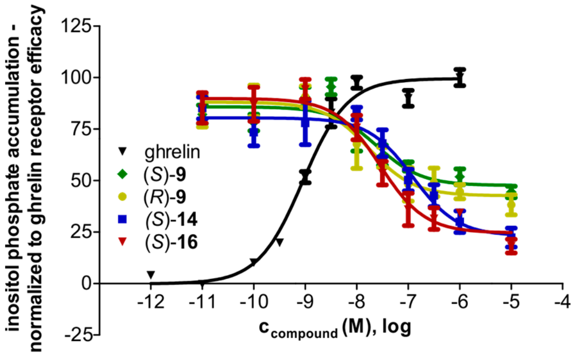

Figure S1. Antagonistic properties of the non-peptidic candidate compounds (*S*)-**9**, (*R*)-**9**, (*S*)-**14**, and (*S*)-**16**. Compounds were analyzed by inositol phosphate accumulation in COS7 cells stably transfected with hGhrR. Data were normalized to ghrelin (100% = maximal efficacy, 0% = constitutive receptor activity) and given in percent as means ± SEM of ≥ 3 independent experiments performed in duplicates.

Selectivity Studies

Table S2. Results of (*S*)-**9** screening by Eurofins Panlabs; for details on the methods see www.eurofinspanlabs.com.

Cat #	Assay Name	Batch*	Spec.	Rep.	Conc.	% Inh.
203100	Adrenergic α_{1A}	395123	rat	2	1 µM	-3
203200	Adrenergic α_{1B}	395160	rat	2	1 µM	9
244530	Chemokine CXCR3	395320	hum	2	1 µM	0
252610	Muscarinic M_1	395145	hum	2	1 µM	14
252710	Muscarinic M_2	395239	hum	2	1 µM	11
265910	Potassium Channel hERG, [^{3}H]Dofetilide	395705	hum	2	1 µM	35
271710	Serotonin (5-Hydroxytryptamine) 5-HT_{2B}, [^{3}H]Mesulergine	395157	hum	2	1 µM	4

1. Kostelnik, K.B.; Els-Heindl, S.; Kloting, N.; Baumann, S.; von Bergen, M.; Beck-Sickinger, A.G. High metabolic in vivo stability and bioavailability of a palmitoylated ghrelin receptor ligand assessed by mass spectrometry. *Bioorg. Med. Chem.* **2015**, *23*, 3925-3932.
